# Complete Genome Sequence of a *Francisella tularensis* subsp. *holarctica* Strain from Germany Causing Lethal Infection in Common Marmosets

**DOI:** 10.1128/genomeA.00135-12

**Published:** 2013-02-07

**Authors:** Markus H. Antwerpen, E. Schacht, P. Kaysser, W. D. Splettstoesser

**Affiliations:** Bundeswehr Institute of Microbiology, Munich, Germany

## Abstract

Here, we describe the genome sequence of the *Francisella tularensis* subsp. *holarctica* strain F92, belonging to the Franco-Iberian subgroup. This strain represents the first-time isolate of this subgroup in Germany and was obtained from naturally infected marmosets.

## GENOME ANNOUNCEMENT

Tularemia is a rare Holarctic zoonosis caused by the Gram-negative, pleomorphic, and facultative intracellular pathogen *Francisella tularensis*. *F. tularensis* is a CDC category A select agent, which may cause several clinical symptoms from localized cutaneous ulcerations at the site of infection and swelling of local lymph nodes to fatal pneumonia or septicemia (1).

Here, we describe the genomic features of the *F. tularensis* subsp. *holarctica* strain F92, which caused fatal tularemia in six naturally infected marmosets (*Callithrix jacchus*) held semifree in a research unit near Göttingen right in the geographical center of Germany (2).

*Francisella tularensis* subsp. *holarctica* F92 was isolated from liver and grown in heart-cystine broth at 37°C and 5% CO_2_ for 48 h. Antibiotic resistances were determined and the phylogeny of the strain was elucidated using actual typing techniques for *Francisella* species, such as single nucleotide polymorphism (SNP) and/or multiple-locus variable-number tandem repeat analysis (MLVA) typing.

For genome sequencing, DNA was extracted, quality was controlled, and reads were generated from 454 GS-FLX sequencing (3). Assembly was done using the GS *de novo* assembler (“Newbler”) version 2.5.3 (Roche Diagnostics). Additionally, three paired-end libraries (0.8 kb, 2 kb, and 30 kb) were generated and sequenced by classical Sanger sequencing. Data of both approaches were used for genome assembly and led to a 44.6-fold overall coverage on average. The resulting contigs were mapped to *Francisella tularensis* subsp. *holarctica* FTA (NC_009749) (4) as a reference genome, resulting in scaffolds with 19 gaps. These gaps were closed by Sanger sequencing of PCR amplicons across the gaps, resulting in a single closed circular chromosome of 1,886,888 bp.

This genome was uploaded to the Rapid Annotation using Subsystem Technology (RAST) website (http://rast.nmpdr.org/rast.cgi) (5). The G+C content was 32.17% (calculated by an in-house python script). A total of 2,190 coding sequences were found, of which 60.1% could be assigned to clusters of orthologous groups (COGs) (6). The NCBI Prokaryotic Genomes Automatic Annotation Pipeline (PGAAP) was employed for gene annotation in preparation for submission to GenBank (http://www.ncbi.nlm.nih.gov/genomes/static/Pipeline).

The genome contains three ribosomal RNAs, four 5S rRNAs, and 38 tRNA loci. Further preliminary analysis of strain F92 showed very similar characteristics (e.g., length, GC content, open reading frames [ORFs], pseudogenes) to all *F. tularensis* subsp. *holarctica* strains recently described (e.g., *F. tularensis* subsp. *holarctica* FTA or OSU18). Most strikingly, we revealed that strain F92 harbored a 1.59-kb deletion (RD23) believed to be specific to *F. tularensis* subsp. *holarctica* strains from France and the Hispanic peninsula (7). Genomic comparison showed that the closest neighbor of strain F92 is strain FTA, differing in only one missing 3,847-nt-long hypothetical gene.

As more strains from this subgroup are investigated, new insights into the microevolution of this pathogenic agent will be gained. This knowledge may help us to understand the epidemiology of this new subgroup of *Francisella tularensis* invading and expanding its geographical distribution from the Hispanic peninsula into Central Europe.

### Nucleotide sequence accession numbers.

This project has been deposited at DDBJ/EMBL/GenBank under the accession no. CP003932. The version described in this paper is the first version, CP003932.1.
